# “This is an illness. No one is supposed to be treated badly”: community-based stigma assessments in South Africa to inform tuberculosis stigma intervention design

**DOI:** 10.1186/s44263-024-00070-5

**Published:** 2024-06-24

**Authors:** Isabel Foster, Amanda Biewer, Nosivuyile Vanqa, Goodman Makanda, Phumeza Tisile, Sally E. Hayward, Dillon T. Wademan, Michaile G. Anthony, Rachel Mbuyamba, Michelle Galloway, Wieda Human, Helene-Mari van der Westhuizen, Jon S. Friedland, Andrew Medina-Marino, Ingrid Schoeman, Graeme Hoddinott, Ruvandhi R. Nathavitharana

**Affiliations:** 1 TB Proof, Cape Town, South Africa; 2 International Development Research Center, Global Health Program, Ottawa, Canada; 3 Beth Israel Deaconess Medical Center and Harvard Medical School, Boston, MA USA; 4 Desmond Tutu TB Centre, Department of Paediatrics and Child Health, Faculty of Medicine and Health Sciences, Stellenbosch University, Stellenbosch, South Africa; 5 Institute for Infection and Immunity, St George’s, University of London, London, UK; 6 Nuffield Department of Primary Care Health Sciences, Oxford University, Oxford, UK; 7 Desmond Tutu HIV Centre, University of Cape Town, Cape Town, South Africa; 8 Desmond Tutu Health Foundation, Cape Town, South Africa; 9 Department of Psychiatry, Perelman School of Medicine, University of Pennsylvania, Philadelphia, USA; 10 School of Public Health, Faculty of Medicine and Health, The University of Sydney, Camperdown, Australia

**Keywords:** Tuberculosis, Stigma, Intervention, Cascade of care, Community-engaged research

## Abstract

**Background:**

Though tuberculosis (TB)-related stigma is a recognized barrier to care, interventions are lacking, and gaps remain in understanding the drivers and experiences of TB-related stigma. We undertook community-based mixed methods stigma assessments to inform stigma intervention design.

**Methods:**

We adapted the Stop TB Partnership stigma assessment tool and trained three peer research associates (PRAs; two TB survivors, one community health worker) to conduct surveys with people with TB (PWTB, *n* = 93) and caregivers of children with TB (*n* = 24) at peri-urban and rural clinic sites in Khayelitsha, Western Cape, and Hammanskraal, Gauteng Province, South Africa. We descriptively analyzed responses for each stigma experience (anticipated, internal, and enacted), calculated stigma scores, and undertook generalized linear regression analysis. We conducted 25 in-depth interviews with PWTB (*n* = 21) and caregivers of children with TB (*n* = 4). Using inductive thematic analysis, we performed open coding to identify emergent themes, and selective coding to identify relevant quotes. Themes were organized using the Constraints, Actions, Risks, and Desires (CARD) framework.

**Results:**

Surveys revealed almost all PWTB (89/93, 96%) experienced some form of anticipated, internal, and/or enacted stigma, which affected engagement throughout the care cascade. Participants in the rural setting (compared to peri-urban) reported higher anticipated, internal, and enacted stigma (β-coefficient 0.72, 0.71, 0.74). Interview participants described how stigma experiences, including HIV intersectional stigma, act individually and together as key constraints to impede care, leading to decisions not to disclose a TB diagnosis, isolation, and exclusion. Stigma resilience arose through the understanding that TB can affect anyone and should not diminish self-worth. Risks of stigma, driven by fears related to disease severity and infectiousness, led to care disengagement and impaired psychological well-being. Participants desired counselling, identifying a specific role for TB survivors as peer counselors, and community education.

**Conclusions:**

Stigma is highly prevalent and negatively impacts TB care and the well-being of PWTB, warranting its assessment as a primary outcome rather than an intermediary contributor to poor outcomes. Multi-component, multi-level stigma interventions are needed, including counseling for PWTB and education for health workers and communities. Such interventions must incorporate contextual differences based on gender or setting, and use survivor-guided messaging to foster stigma resilience.

**Supplementary Information:**

The online version contains supplementary material available at 10.1186/s44263-024-00070-5.

## Background

In 2022, of the 10.6 million people who became sick with tuberculosis (TB), 280,000 were in South Africa, which is one of 30 high TB incidence countries [1]. TB stigma is a recognized barrier to care, causing delays in care-seeking and treatment initiation, and gaps in engagement and adherence [2–6]. Stigma manifests in different forms including enacted stigma through the negative actions of others, anticipated stigma based on the expected negative actions of others, and internal stigma arising from negative actions or beliefs towards oneself [7–9]. Strategic documents such as the World Health Organization’s (WHO) End TB strategy and the United Nations High-Level Meeting on TB include stigma reduction as an important strategy to decrease TB transmission and disease burden [10, 11]. Yet, evidence to inform the design and implementation of TB stigma interventions is limited [12, 13].

Despite guidance on TB stigma measurement [14], existing stigma intervention studies use heterogeneous approaches to define and measure stigma experiences, and most do not have stigma reduction as a primary outcome [12]. An exploratory analysis of TB stigma within a contact tracing study in South Africa illustrates how individuals with TB are marked by TB stigma, resulting in a reluctance to engage with facility and home-based services [4]. Other data from South Africa highlight how TB care engagement is affected by gender and community social standing, both of which are linked to stigma [15]. There remain major gaps in our understanding of TB stigma, its drivers, its differential effects on groups, and its impact on outcomes.

The cascade of care model quantifies gaps in the retention of people with TB (PWTB) at each stage of care from care-seeking to diagnosis to treatment and cure, and has shown that only about 50% are estimated to complete treatment [16]. A care cascade analysis for South Africa reported that 25% of the estimated 532,005 PWTB are lost between accessing diagnostic testing and initiating treatment, and 19% are lost between treatment initiation and completion [17], underlining the need to improve care engagement. Human immunodeficiency virus (HIV) research has shown that stigma is not experienced uniformly across the care cascade [18]. The effects of stigma across the TB cascade are less well characterized but anticipated and internal stigma appears to contribute to care-seeking delays and loss of follow-up [4, 19]. Understanding how TB stigma manifests according to stages of care is essential to designing effective, evidence-based interventions.

Mixed methods approaches can provide a fuller understanding of complex care engagement and how this maps to measurable program targets than qualitative or quantitative data alone [20, 21]. The use of qualitative research has provided key insights into TB care as a networked phenomenon between actors, including patients and clinicians, and resources, including technologies and health systems, that inform care improvement interventions [22]. Community-engaged research is an established approach to enable meaningful and equitable research participation by affected communities [23]. The use of peer research associates (PRAs), who have lived experiences of the diseases being studied, has been successfully implemented for HIV [24–26], with various benefits including strategies to address recruitment challenges, supporting the engagement of underserved communities, fostering co-production of new knowledge, and enabling translation and dissemination of findings [27]. However, PRAs remain an underexplored opportunity for people affected by TB to engage with, inform, and contribute to research. With the overarching goal of designing and implementing a contextually relevant stigma reduction intervention, we employed a community-engaged mixed methods research approach, co-led by TB survivor PRAs, to understand how, when, and where stigma is experienced by people affected by TB.

## Methods

### Study design and setting

We conducted a mixed methods study with a convergent design, meaning that quantitative and qualitative data were collected and analyzed in a similar timeframe [28]. From January 2021 to March 2022, we undertook community-based stigma surveys and in-depth interviews with people affected by TB. Participants were recruited from two communities located around purposively selected clinic sites in geographically distant provinces in South Africa: (1) Luvuyo Clinic in Khayelitsha, City of Cape Town Health District, Western Cape Province, and (2) Hammanskraal Clinic in Hammanskraal, Tshwane Health District, Gauteng Province. We sought to have one peri-urban site with a higher incidence than the national average (428 per 100,000 population) and one rural site with a lower incidence. In 2019, TB incidence was 527 per 100,000 population in the City of Cape Town district (> 99% Black African, predominantly isiXhosa speaking population) and 181 per 100,000 population in the Tshwane District (98% Black African, predominantly Sotho speaking population) [29]. This community-based participatory research project engaged persons with lived experience of TB as PRAs following the framework of Kaida et al. [27]. The two PRAs (GM and PT) were existing TB survivor advocates working with TB Proof, a South Africa-based TB advocacy organization that co-led this research, and were chosen based on their deep knowledge of the study community and ability to create rapport with participants. PRA training focused on conducting surveys and interviews was provided using a hybrid learning environment including two remote training sessions and two in-person coaching sessions from experienced qualitative researchers (GH, NV, DTW). PRAs (GM and PT) were involved at all stages from project conception to analysis, through dissemination of findings.

### Study population and sampling recruitment

We sought to recruit people who were aged ≥ 18 years and defined as (1) PWTB based on a current or prior diagnosis of TB (not time-limited), or (2) caregivers who had cared for a child (< 18 years) with TB. Survey participants were identified by community health workers (CHWs) working at either of the selected study clinic sites and recruited using convenience sampling. We aimed to enroll approximately 200 survey participants but since the purpose of this study was to descriptively analyze stigma experiences, we did not undertake a power calculation. From the participants who consented to and completed the quantitative survey, we purposively selected participants for in-depth interviews (IDIs). Purposive sampling sought to ensure representation of (1) gender, (2) TB type (drug-susceptible or resistant; pulmonary or extra-pulmonary), and (3) HIV status.

### Stigma measurement tools

We adapted the validated Stop TB Partnership TB Stigma Assessment Tool [30] based on a literature review and iterative feedback from our PRAs who pre-tested the survey. The survey measured anticipated (17 items), enacted (17 items for PWTB, 18 items for caregivers), and internal (16 items) stigma (Additional file 1: Table S1). Measurements were made using a five-level Likert scale (‘strongly agree’, ‘agree’, ‘neutral’, ‘disagree’, and ‘strongly disagree’). These included questions to ascertain how different stigma experiences affected each stage of the care cascade from care-seeking, to diagnosis, to treatment initiation, completion, or cure. We designed a semi-structured IDI guide (Additional file 2: Participant Interview Guide) to cover three key topics: *(a) the person’s TB illness narrative, (b) details about their experiences relating to anticipated stigma, enacted stigma, and internal stigma, and (c) recommendations for interventions to reduce stigma*. The PRAs pre-tested the IDI guide and developed specific examples of (i) internal, (ii) anticipated, and (iii) enacted stigma based on personal experiences to illustrate these concepts, and to establish rapport and make participants feel at ease during IDIs.

### Data collection

Two PRAs (GM, PT) administered the survey in Khayelitsha, and one CHW administered the survey in Hammanskraal. Survey data were collected and managed using Research Electronic Data Capture (REDCap) tools, hosted at Stellenbosch University [31]. Survey participants did not receive reimbursement but were entered into a raffle to win a small prize (worth R500 ≈ $26). IDIs, lasting 30–60 min, were conducted by trained interviewers (PRAs: GM and PT, with support from researcher NV) at either the participant’s home or their local health clinic, and audio recorded. IDIs were conducted either in-person or via telephone. Field notes were written following each IDI. IDI participants received reimbursement for their time and transport (R100 ≈ $5.25). Surveys and IDIs were conducted in the participants' preferred language (isiXhosa in Khayelitsha; Sotho in Hammanskraal).

### Analyses

#### Quantitative

Descriptive statistics were used to describe socio-demographic and clinical survey data. We reported the proportions of participants who selected to agree or strongly agree to at least one or more than five survey items describing experiences of anticipated (11 items), internal (10 items), and enacted (13 items) stigma [32]. We stratified data by gender and compared the proportions of men and women who agreed or strongly agreed with questions about specific stigma experiences using the chi-squared test. We generated stigma scores for each stigma domain by summing the results for each item out of 5 with higher scores indicating higher stigma (minimum score 1 and maximum score 5). Good internal reliability of scales was a priori defined as a Cronbach's alpha of ≥ 0.7. The stigma scales in this study were internally consistent: Cronbach’s alpha was 0.904 for anticipated stigma, 0.897 for enacted stigma, and 0.880 for internal stigma in those who experienced TB. We used linear regression to investigate the determinants of anticipated, enacted, and internal stigma. Based on univariate analyses, we included variables with *p* ≤ 0.1 for inclusion in the multivariate models. Beta-coefficients (β coef) were presented with 95% confidence intervals. A 2-tailed *p* value < 0.05 was considered statistically significant. Statistical analyses were conducted using STATA 16 (StataCorp LP, College Station, TX, USA).

#### Qualitative

IDI audio files were password-encrypted and saved using a unique study ID for each participant prior to transcription and translation into English using professional transcription services, and any potentially identifying information, including names and locations, in the transcripts, was redacted. We completed the COnsolidated criteria for REporting Qualitative research (COREQ) checklist (Additional file 3: COREQ checklist). Transcripts were imported into NVivo 12 software (Lumivero, CO, USA). Two coders (IF, RRN) performed open coding to inductively identify emergent themes related to stigma experiences [33]. No new themes were derived after 25 IDIs, achieving data saturation [34]. We then applied a deductive thematic analysis by categorizing themes according to the Constraints, Actions, Risks, and Desires (CARD) framework [35], which integrates data on individual and systemic factors in a single analytical frame to inform intervention design. We used the CARD framework to analyze the interaction of factors within the networked ecosystem in which PWTB and caregivers contextualize and interpret their perspectives and behaviors related to TB stigma.

Quantitative and qualitative data [36] were analyzed separately, and we triangulated the findings as part of our integrated mixed methods analysis.

## Results

### Stigma survey results for PWTB

#### Survey population characteristics

Surveys were administered to 117 individuals: 93 (79.5%) PWTB and 24 (20.5%) caregivers. The median age of PWTB was 40 years (IQR 33–49), approximately half were women (53%), and 55 (59%) PWTB reported that they were living with HIV (Table 1). The majority of PWTB had pulmonary TB (PTB) (91%) and 9% had drug-resistant TB (DR-TB). Over one-third (37%) of PWTB reported prior TB (Table 1). For caregiver data, please see Additional file 4: Stigma survey results for caregivers and Additional file 5: Table S2.

**Table 1:** Study participant characteristics

	People with TB (*n* = 93)
Demographic variables	
Median age (years, IQR)	40 (33–49)
Gender, *n* (%)	
Woman	49 (53)
Man	44 (47)
Language, *n* (%)	
English	10 (11)
Xhosa	37 (40)
Sotho	45 (48)
Missing	1 (1)
Location, *n* (%)	
Khayelitsha	47 (51)
Hammanskraal	45 (49)
Missing	1 (1)
Clinical variables	
HIV, *n* (%)	
Yes	55 (59)
No	18 (19)
Refused	9 (10)
Missing	11 (12)
TB type, *n* (%)	
Pulmonary	85 (91)
Extra-pulmonary	8 (9)
Drug resistance type, *n* (%)	
Drug sensitive	85 (91)
MDR or XDR	8 (9)
Previous TB, *n* (%)	
Yes	34 (37)
No	59 (63)

*Abbreviations*: *IQR* interquartile range, *MDR* multidrug resistant, *XDR* extensively drug resistant

#### Stigma scores, experiences, and settings

Stigma scores were not normally distributed. The median and interquartile range (IQR) was 2.57 (2.08–3.05) for anticipated stigma, 2.44 (2.06–3) for internal stigma, and 2.16 (1.84–2.80) for enacted stigma (Table 2). Almost all PWTB reported experiencing some form of anticipated, internal, or enacted stigma, with 96% (89 of 93) reporting agreeing or strongly agreeing to at least one item measuring these forms of stigma (Table 2). Anticipated and enacted stigma were more commonly experienced by PWTB in their communities (65% and 48%) than within their families (56% and 30%) respectively. For caregiver data, please see Additional file 6: Table S3.

**Table 2:** Stigma scores, experiences of stigma, and settings where these occurred

	PWTB (*n* = 93)
*Stigma scores*	
Anticipated stigma (median, IQR)	2.57 (2.08–3.05)
Internal stigma (median, IQR)	2.44 (2.06–3)
Enacted stigma (median, IQR)	2.16 (1.84–2.8)
*At least 1 survey item with an agree/strongly agree response*	
Anticipated stigma (11 items), *n* (%)	89 (96)
Internal stigma (10 items), *n* (%)	89 (96)
Enacted stigma (11 items), *n* (%)	89 (96)
*At least 5 items with an agree/strongly agree response*	
Anticipated stigma (11 items), *n* (%)	67 (72)
Internal stigma (10 items), *n* (%)	40 (43)
Enacted stigma (11 items), *n* (%)	52 (56)
*Settings of stigma experiences*	
Anticipated stigma-community	
Yes	53 (65)
No	29 (34)
Neutral	1 (1)
Anticipated stigma-family (or friends)	
Yes	51 (56)
No	40 (43)
Neutral	1 (1)
Enacted stigma-community	
Yes	42 (48)
No	39 (46)
Neutral	5 (6)
Enacted stigma-family (or friends)	
Yes	28 (30)
No	63 (68)
Neutral	2 (2)

*Abbreviations*: *IQR* interquartile range

#### Impacts of anticipated, internal, and enacted stigma on engagement throughout the care cascade

**Fig. 1:**
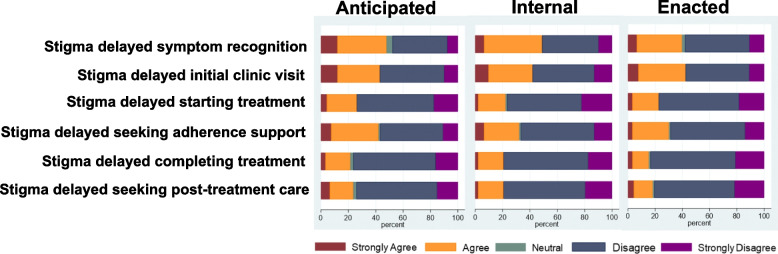
Responses from people with TB regarding the effects of anticipated, internal and enacted stigma on care cascade engagement. The horizontal axis of the bar shows the percentages of respondents who strongly agreed, agreed, disagreed, and strongly disagreed with the statements used in the scale to measure each type of stigma experience

**Fig. 2:**
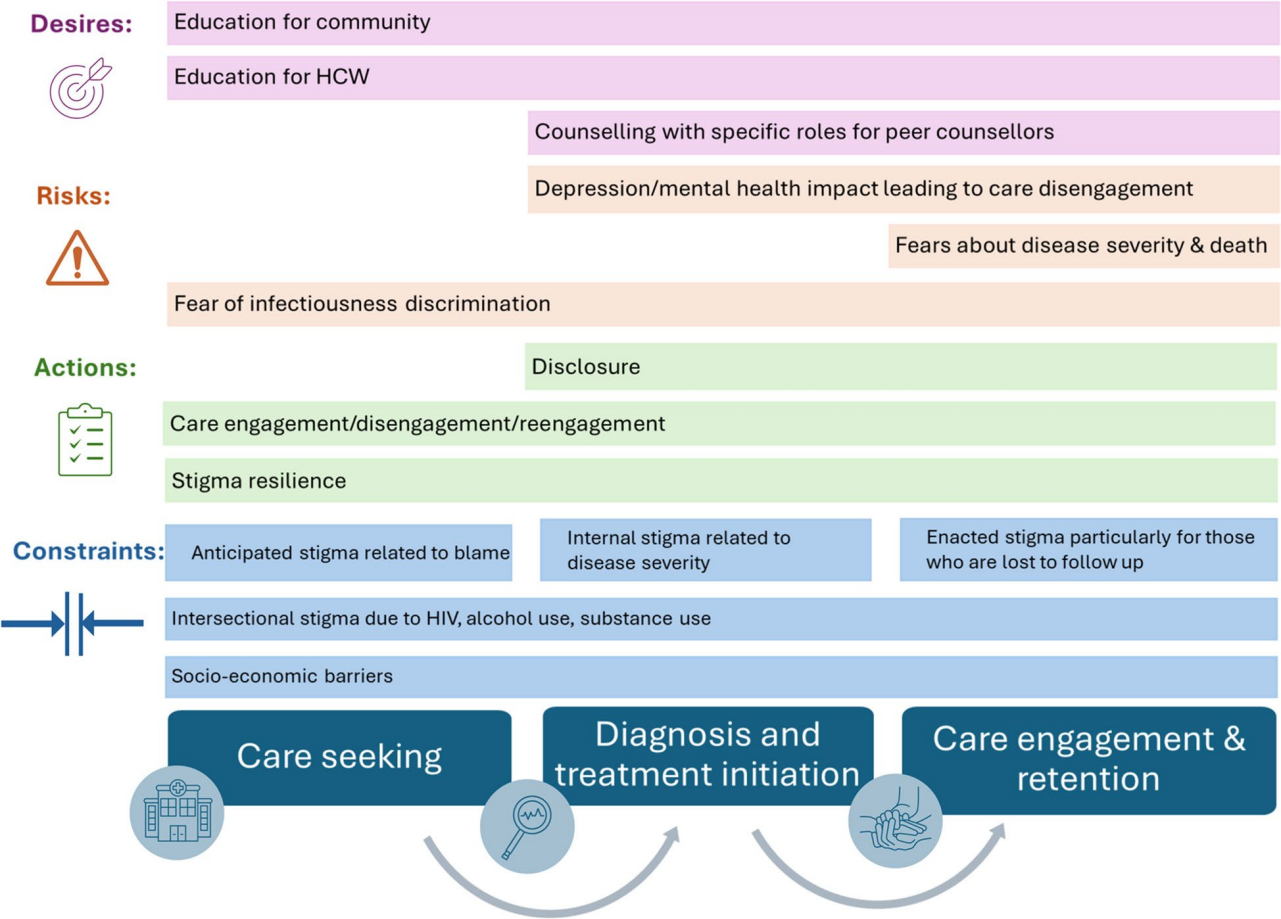
Conceptual figure illustrating how the themes identified across CARD domains occur across the TB cascade of care

A high proportion (between 20 and 40%) of PWTB strongly agreed or agreed that experiences of all three types of stigma impacted care engagement at sequential steps of the cascade. Importantly experiences of stigma continued to be frequent after PWTB have already engaged with care, which presents an opportunity for care services to improve the care of existing patients (Figs. 1 and 2. For caregiver data, please see Additional file 7: Figure S1.

#### Living in a rural area, compared to a peri-urban setting was associated with increased stigma

Univariate linear regression analyses demonstrated increased scores for anticipated, internal, and enacted stigma experienced by PWTB in Hammanskraal and those who reported prior TB (Table 3). Univariate analyses suggested that extra-pulmonary TB was associated with lower internal, and enacted stigma scores and that DR-TB was associated with lower internal stigma scores. Gender and HIV were not associated with differences in stigma scores. We included age, location, prior TB history, TB type, and drug resistance pattern in the multivariate models. Only living in Hammanskraal (rural area) compared to Khayelitsha (peri-urban area) was associated with higher scores in the multivariate model: internal stigma (β coef 0.71; 95% CI 0.40, 1.03; *p* < 0.001), anticipated stigma (β coef 0.72; 95% CI 0.46, 0.97; *p* < 0.001) and enacted stigma (β coef 0.74; 95% CI 0.50, 0.97; *p* < 0.001) (Table 3). For caregiver data, please see Additional file 8: Table S4.

**Table 3:** Factors associated with higher stigma scores reported by people with TB

	Anticipated stigma				Internal stigma				External stigma			
Variable	Crude β-coef (95% CI)	*p* value	Adjusted β-coef (95% CI)	*p* value	Crude β-coef (95% CI)	*p* value	Adjusted β-coef (95% CI)	*p* value	Crude β-coef (95% CI)	*p* value	Adjusted β-coef (95% CI)	*p* value
Age categories, *n* (%) in years												
< 30 31–40 41–50 51–60 > 60	*Reference* **0.41 (0.05, 0.78)** 0.38 (− 0.01, 0.76) 0.06 (− 0.39, 0.51) − 0.27 (− 0.92, 0.37)	– **0.027** 0.054 0.798 0.404	*Reference* − 0.03 (− 0.38, 0.31) − 0.01 (− 0.37, 0.35) − 0.01 (− 0.41, 0.39) − 0.15 (− 0.77, 0.47)	– 0.860 0.960 0.958 0.627	*Reference* 0.32 (− 0.14, 0.77) 0.15 (− 0.33, 0.62) 0.03 (− 0.53, 0.59) − 0.24 (− 1.04, 0.57)	– 0.168 0.543 0.916 0.561	*Reference* − 0.22 (− 0.64, 0.20) − 0.28 (− 0.72, 0.15) − 0.10 (− 0.59, 0.39) − 0.15 (− 0.91, 0.61)	– 0.308 0.197 0.683 0.690	*Reference* 0.31 (− 0.03, 0.66) 0.24 (− 0.13, 0.60) − 0.11 (− 0.55, 0.32) − 0.25 (− 0.87, 0.37)	– 0.076 0.208 0.602 0.429	*Reference* − 0.18 (− 0.49, 0.14) − 0.20 (− 0.52, 0.13) − 0.23 (− 0.60, 0.13) − 0.19 (− 0.66, 0.38)	– 0.266 0.233 0.201 0.513
Gender, *n* (%)												
Woman Man	*Reference* − 0.03 (− 0.27, 0.22)	– 0.837	–	–	*Reference* − 0.09 (− 0.30, 0.12)	– 0.388	–	–	*Reference* − 0.18 (− 0.41, 0.05)	– 0.121	–	–
HIV, *n* (%)*												
Yes No	*Reference* − 0.09 (− 0.28, 0.10)	– 0.354	–	–	*Reference* − 0.08 (− 0.37, 0.22)	– 0.609	–	–	*Reference* − 0.15 (− 0.33, 0.04)	– 0.113	–	–
History of Previous TB, *n* (%)												
Yes No	*Reference* **0.31 (0.06, 0.55)**	– **0.016**	*Reference* 0.05 (− 0.18, 0.28)	– 0.680	*Reference* **0.53 (0.25, 0.81)**	– ** < 0.001**	*Reference* 0.27 (− 0.02, 0.55)	– 0.067	*Reference* **0.33 (0.10, 0.56)**	– **0.006**	*Reference* 0.11 (− 0.10, 0.33)	– 0.288
TB type, *n* (%)												
Pulmonary Extrapulmonary	*Reference* − 0.33 (− 0.77, 0.10)	– 0.128			*Reference* **− 0.55 (− 1.06, − 0.04)**	– **0.035**	*Reference* – 0.01 (– 0.47, 0.49)	– 0.965	*Reference* **− 0.45 (− 0.86, − 0.05)**	– **0.030**	*Reference* − 0.06 (− 0.42, 0.30)	– 0.734
Drug resistance, *n* (%)												
Drug sensitive DR-TB	*Reference* − 0.43 (− 0.85, 0.004)	– 0.052	*Reference* − 0.07 (− 0.47, 0.33)	- 0.736	*Reference* **− 0.81 (− 1.3, − 0.32)**	– **0.002**	*Reference* − 0.33 (− 0.82, 0.16)	– 0.184	*Reference* –0.38 (–0.79, 0.03)	– 0.068	*Reference* 0.09 (− 0.27, 0.46)	– 0.620
Location,*n* (%)												
Khayelitsha Hammanskraal	*Reference* **0.73 (0.53, 0.92)**	– **< 0.001**	*Reference* **0.72 (0.46, 0.97)**	- **< 0.001**	*Reference* **0.77 (0.52, 1.01)**	– **< 0.001**	*Reference* **0.71 (0.40, 1.03)**	– **< 0.001**	*Reference* **0.73 (0.56, 0.91)**	– **< 0.001**	*Reference* **0.74 (0.50, 0.97)**	– **< 0.001**

#### Gender is associated with different experiences of each type *of stigma*

While stigma measure scores did not differ when stratified by gender, examination of responses to individual items revealed differences (Table 4). Compared to men, women were more likely to report anticipated stigma related to fears about gossip or losing friends. Women were more likely to report internal stigma due to their feeling that they should be kept away from other people, whereas men were more likely to feel internal stigma due to the association of TB with behavioral risk factors (i.e., alcohol use or smoking). Similarly, women were more likely to report enacted stigma due to people talking badly about them, losing respect in the community, or being undermined by people due to having TB, whereas men were more likely to report enacted stigma due to the association others made with stigmatized behaviors such as drinking or smoking (Table 4).

**Table 4:** Stigma survey items for which responses differed between women and men with TB

Stigma survey statements	Proportion of women who strongly agreed/agreed	Proportion of men who strongly agreed/agreed	*p* value
Anticipated stigma (11 items examined)			
I have worried that people might talk badly/gossiped about me because I had TB	21/49 (43%)	9/44 (20%)	**0.021**
Internal stigma (10 items examined)			
I have felt that having TB means I should be kept away from other people (alone)	23/49 (47%)	11/44 (25%)	**0.028**
I probably got TB because I smoke, drink, or do other careless things	13/49 (27%)	22/44 (50%)	**0.020**
External stigma (12 items examined)			
People have talked badly/gossiped about me because I had TB	39/49 (80%)	26/44 (59%)	**0.031**
I have lost respect/standing in the community because I had TB	34/49 (69%)	15/44 (34%)	**0.001**
I have been undermined by people because I had TB	31/49 (63%)	16/44 (36%)	**0.010**
Someone has told me that I have TB because I smoke, drink, or do things that other people might think of as 'careless'	10/49 (20%)	18/44 (41%)	**0.031**

### In-depth interview findings

We interviewed 21 PWTB (14 women, seven men): eight of whom had DR-TB and six of whom had HIV, and four caregivers (who took care of three boys and one girl): one of whom had HIV. Most interview participants lived in Khayelitsha (22); three lived in Hammanskraal. Key findings are presented according to the domains of the CARD framework.

#### Constraints: stigma, including intersectional stigma, is a major barrier to TB care and is often linked with other socioeconomic barriers

Participants commonly experienced anticipated stigma, due to fears that friends or community members would see them engaged in TB care leading to reputational impacts. Enacted stigma included loss of friends after being diagnosed with TB and discrimination by health workers. Internal stigma was sometimes linked to physical changes like weight loss that signified disease to others, and also to intersectional stigma related to HIV, smoking, and alcohol, as risk factors for TB.

“When a person is looking at you, it is as if they are feeling pity for you. They think they are better, that they will never be infected with TB.” (016, man, MDR-TB, Hammanskraal).

Socio-economic constraints, sometimes linked to stigma, including the loss of employment attributed to TB and food insecurity, were frequently mentioned, along with limitations of the existing disability grants from the South African Social Security Agency (SASSA) program [37].

“[My life] changed because I'm unemployed. I'm struggling in life because I must live with SASSA and I can't do the things I used to do even now [post TB]. With this SASSA the food gets finished quickly because I must also feed other people.” (003, woman, MDR-TB, Khayelitsha).

#### Actions: stigma negatively affected behaviors related to TB diagnosis, disclosure, and care (re)engagement, although examples of stigma resilience were apparent

Internal stigma was a driver of delayed care seeking, and feelings of negative self-worth persisted beyond TB treatment. Anticipated stigma affected care engagement throughout the cascade, and was a major driver for participants deciding not to disclose their TB diagnosis, particularly for men, leading to feelings of isolation, a lack of support, and a negative impact on social relationships.

“He might end up not coming to clinic. When he witnesses those who are marginalised, he might end up being afraid to come to clinic because he thinks he will be marginalised.” (012, man, Khayelitsha).

Experiences of enacted stigma from health workers affected participants’ decisions about seeking and/or remaining engaged in care. Participants described being particularly vulnerable during hospitalization and in the first weeks of TB treatment and noted that enacted stigma was compounded for PWTB who were re-engaging with care after treatment interruptions. To avoid HIV intersectional stigma, some participants actively showed friends or family that their medications were for TB and not for HIV.

“I showed [my friends] that I take treatment for TB and it’s not for AIDS [laughs]. They were thinking that I am taking treatment for AIDS and hiding it from them.” (002, woman, Khayelitsha).

Some participants described beliefs and attitudes that appeared to make them resilient to stigma. This resilience often appeared to arise from understanding TB can affect anyone, which resulted in avoiding self-blame and expecting that they should not be treated differently (or worse) than people without TB. Participants who disclosed their TB status often mentioned practical reasons or benefits for doing so, including logistical and emotional support, which served as strong enablers to treatment completion.

“This is an illness. No one is supposed to be treated badly because a person didn't ask to have TB. A person is supposed to be treated the same. Don’t look down on them.” (001, woman, Khayelitsha).

#### Risks: TB diagnosis was associated with negative mental health impacts that led to care disengagement, and fears related to infectiousness leading to self and social exclusion

TB and associated stigma often had a negative impact on PWTB’s mental health, with several reporting depression explicitly. Participants mentioned being in denial about their symptoms or illness. Several reported self-isolation, linked to internal stigma, which also contributed to care disengagement.

“I didn’t even know it was depression because I didn’t even want to cooperate.” (024, woman, XDR-TB, Khayelitsha).

Internal stigma also arose due to fears about the risk of severe illness associated with TB, leading to feelings of hopelessness, compounded in people with DR-TB. At least one participant linked their fears to the pervasive belief in communities that the inevitable prognosis for TB is death. Anticipated and enacted stigma, driven by fears of infectiousness, fuelled concerns about posing risks to others. This resulted in PWTB isolating themselves from others and being excluded. Misperceptions about infectiousness despite being on treatment acted as a stigma driver and led to further isolation of PWTB.

“They are afraid to be infected by a person who has TB. But they don't have the knowledge about TB, because when you are taking the treatment, it's not contagious.” (020, woman, Khayelitsha).

#### Desires: participants suggested that internal and anticipated stigma could be mitigated by offering counselling for PWTB and families, and anticipated and enacted stigma could be reduced by training health workers on stigma and community-wide education about stigma

Desires related to reducing stigma and improving the well-being of PWTB and TB-affected communities fell into three categories: counseling for PWTB and their families, stigma reduction training for health workers, and education for communities to close knowledge gaps and decrease stigma. Poor counseling was highlighted as an important barrier to person-centered TB care. Peer counseling, with exposure to the lived experiences of TB survivors, was recommended. Efforts to reduce enacted stigma by health workers were thought to be particularly important when addressing people who interrupt treatment or are lost to follow-up (still commonly referred to as ‘defaulters’), with the goal of ensuring people are not judged or discriminated against. Health worker stigma training, informed by patient perspectives, could improve their understanding of the challenges experienced by PWTB and enhance their care delivery.

“And at the hospital, a person is shouted at by the nurses that they don't take their pills: “That's why you default". That name came to me, that I defaulted.” (018, woman, MDR-TB, Khayelitsha).

Participants mentioned the lack of broader TB knowledge within the community. This was seen as a driver of stigma and poor care engagement. Namely, participants emphasized the importance of ensuring communities have accurate information to understand how TB can affect anyone, how TB is spread, and how long PWTB remains infectious to others after treatment.

### Triangulation of quantitative and qualitative findings

IDIs provided explanatory data on the negative effects of stigma experiences measured in the quantitative surveys. Anticipated and internal stigma, emanating from the community often related to intersectional stigma and fears of infectiousness, resulted in hesitation to seek care. Enacted stigma from community members and health workers affected retention in care. Some of the gender-based differences in stigma experiences noted in the surveys were corroborated by IDIs. For example, changes in appearance and status affect women’s psychological well-being but may not prevent them from engaging in care. In comparison, discrimination due to intersectional stigma associated with alcohol use and smoking led to men feeling ashamed and less likely to engage in care. While HIV was not associated with higher stigma scores, IDI participants often mentioned HIV intersectional stigma. The low number of IDI participants from Hammanskraal limited the exploration of factors that may explain why stigma scores were higher among survey participants in that setting.

### Example stigma intervention components based on mixed methods stigma assessment findings

Table 5 uses the CARD framework [35] to organize the findings of our mixed methods stigma assessments. We report illustrative quotations and insights for stigma intervention design, including example stigma intervention components that were identified by the research team, including the PRAs.

**Table 5:** Use of the CARD analytical framework to design stigma interventions informed by community-based stigma assessments

Card domains and themes	Illustrative quotations	Researcher recommendations for applying components to a stigma intervention
Constraints		
– Internal stigma – Anticipated stigma – External stigma	“I told myself that people who can get TB are smokers, people who work in cold places. I never thought that I would get TB.” "What is so and so going to say when I'm on the side of people with TB, who are taking the TB treatment?" “There were no people coming to my house when I was sick”	Individual counseling for all TB patients is needed, including messaging that TB can happen to anyone and should not be seen as an indictment of someone’s worthiness, and discussion about how to address the anticipated and external stigma that may arise
Constraints – Intersectional stigma: HIV, alcohol use, gender	“If you lose weight then they assume that you have HIV. They would say you might have HIV and you should go and get tested.” “He was drinking alcohol and he died.” “Us man people, we are worse. We are embarrassed to go to the clinic and take treatment”	Integrated TB/HIV stigma counseling and targeted education campaigns can educate communities on risk factors for TB and reduce intersectional stigma The use of risk screening strategies can help identify people with alcohol or substance use at risk of poor outcomes, alongside messaging that anyone can develop TB Gender-tailored care engagement approaches should address differences in stigma for men and women
Constraints – Socio-economic barriers and exacerbation of these due to TB	“I struggled when it comes to food and I am still struggling now because I am not working.” "I don't have money, I don't have anything to eat. If I take these pills, they make me sick"	Interventions should incorporate components such as food or transport vouchers Information and assistance with obtaining social support should be available to TB patients
Actions – Disclosure decisions varied depending on anticipation and experiences of enacted stigma	“[On the topic of telling friends about their TB] I knew that they were going to gossip about me.” “No! I never had any problem at all with telling them, I was free, free, free, they also know, they used to help me, my family encouraged me”	Interventions should include members of a PWTB’s social network, such as family-centered counseling (for example family workshops and home visits to strengthen individual support networks)
Actions – Stigma resilience demonstrated by understanding TB can affect anyone	“I didn't fear anything when it came to that [decision to seek healthcare], I just thought everyone can be infected by TB.” “I was telling her that she [child with TB] is not the only one that has TB. There are a lot of people that have TB.”	Education on the universality of the risk of TB infection can support de-linking TB infection with blame for persons infected Support groups among PWTB can facilitate co-learning from disclosure experiences
Risks – Depression – Care disengagement more likely due to impacts on mental health	“I had nightmares. Sometimes I had a dream of being buried. Maybe if I knew how depression starts, maybe I would say it was starting because there was a time where I didn’t want to be around people, I wanted to be alone. And sometimes, I didn't even take those pills.” “Once a person has started treatment, we go around talking about them, saying that "So and so has TB". This person is even afraid to leave the house, to go to the clinic.”	Support groups among PWTB can facilitate co-learning and knowledge sharing on coping mechanisms to mitigate the risk of depression Integration of screening and referral for mental health conditions into routine TB care can further ensure that PWTB is supported
Risks – Disease severity	‘People don’t have hope. When people talk about TB, they would say that a person will die or say a person won't survive. When I heard a certain person has died, and they had TB, I had that fear within me that "Wow! I also have TB"	Counseling mechanisms including support groups and connection with TB survivors can help ensure people with TB are better informed on the outcomes of TB treatment and survival rates
Risks – Fear of infectiousness and transmission	“People who were my friends, who I used to help, isolated themselves from me.” “I think he [child with TB] was also scared to go play with them since people say that TB is infectious.”	Family workshops to provide education at the household level and community education interventions can support better understanding at the interpersonal and community level on infectiousness and transmission
Desires – Counselling, including specific roles for peer counselors	“I would say that they [counsellors] are needed, but should be someone who has experience of what you are dealing with, who has felt the pain you would be feeling like the side effects.”	Counseling mechanisms including support groups and connection with TB survivors can help to ensure that people with TB feel supported as they undergo treatment
Desires – Education for health workers and communities	I said to the doctor "You educated people have a name saying ‘default’. You're mistaken. I didn't default, I'm just not well" “I listen a lot to the things being taught at the clinics. We could have a venue where people will be invited to be taught about TB.”	Educational campaigns can reduce misinformation and associated stigma, for example, targeted education and training for health workers or community campaigns can reduce stigma

## Discussion

Community-based surveys with PWTB in two settings in South Africa reveal high levels of anticipated, internal, or enacted TB stigma, which negatively affected engagement throughout the care cascade. Stigma scores were higher in the rural community compared to the peri-urban community. Analysis of IDIs using the CARD framework identified constraints, actions, risks, and desires that inform stigma intervention design [35]. Anticipated, internal, and enacted stigma, including HIV intersectional stigma, act as key constraints, often compounded by socioeconomic barriers, to affect care engagement. Specifically, stigma experiences negatively affected actions such as decisions about care seeking, engagement, and disclosure, although some participants displayed beliefs and attitudes that fostered stigma resilience. Risks linked to stigma included loss of follow-up and long-term impacts on mental health and well-being. Participants desired counseling and educational interventions to reduce stigma in families, communities, and health systems.

Our findings demonstrate how stigma negatively affects decisions and behaviors surrounding care engagement and disclosure. The high levels of stigma that continue to occur after people engage with TB care services underscore the potential impact of stigma interventions following TB diagnosis to reduce treatment interruption. Another cross-sectional study conducted in South Africa demonstrated higher stigma in presumptive patients compared to those post-diagnosis [19], which suggests the importance of dispelling stigma through community-focused interventions [38]. Prior TB stigma intervention studies have typically been pilot phase and only focused on one socioecological level [12, 13], yet our data indicate the need for multi-level interventions to address different stigma experiences across the cascade [39]. Moreover, given its impact on care outcomes and longer-term well-being, we recommend that TB-related stigma should be evaluated and measured as a primary indicator rather than merely as a programmatic or medicalized indicator of the TB cascade [12]. We note that eliminating stigma, as a social determinant of health [40], differs from interventions like diagnostic tests or regimens, as it is intrinsically linked to other complex issues including poverty and racism [41–43], but contend that measuring stigma and evaluating how interventions affect stigma is critical.

Similarly to our findings, increased TB stigma in rural areas compared to urban areas has been reported [19, 44], although other studies show higher or similar levels of stigma in urban areas [44, 45]. The higher TB incidence in our peri-urban compared to rural settings may be associated with greater awareness of TB, which may influence stigma. We nonetheless acknowledge that differences in stigma experiences between study communities may reflect the different ethnic groups and their cultural norms rather than necessarily the rural versus urban context. HIV intersectional stigma was often mentioned, which others have described in the context of TB and poverty [46]. HIV prevalence is higher in Tshwane compared to Cape Town, which may also increase stigma [47]. Triangulation of our quantitative and qualitative findings provides insight into how and why women and men appear to experience stigma differently, which others have also reported [38, 48]. Interventions should be tailored to address drivers of anticipated and enacted stigma related to changes in physical appearance that may lead to social isolation of women and intersectional stigma related to alcohol and substance use that may lead to social exclusion of men [38, 49]. By intervening on stigma drivers, stigma can be interrupted prior to its occurrence, rather than post-occurrence when interventions serve to mitigate its effects [50].

Stigma resilience was associated with PWTB feeling empowered to disclose their illness to their families, which then opened up access to mental and tangible resources to support their care journey. A study from Ethiopia found that decisions surrounding TB disclosure are strongly linked to anticipated stigma [51]. Studies from South Asia also show that lower levels of stigma facilitate the disclosure and investigation of non-household contacts [52–54]. Strategies to reduce HIV stigma such as considering personal rather than only public health motivation to facilitate disclosure conversations [55] could be adapted, along with eliminating criminalizing and discriminatory terms, such as suspect or defaulter [18]. PWTB in our study who understood that TB can affect anyone and did not link TB to their self-worth appeared more resilient to stigma, highlighting potential messaging for counselling interventions.

Our study participants emphasized the role that TB survivors could play to improve care delivery and engagement at various cascade steps by sharing their experiences, insights, and offering support. While there are numerous examples of the use of peer navigators including people with HIV to improve care engagement, published data on TB survivor peer support interventions are sparse [56–59], although findings from other studies point to the need for TB peer navigators [48, 60]. Although counseling is a recommended component of TB care delivery [49], we identified major gaps in its implementation, aligned with other studies from South Africa [42, 61, 62]. Our data support systematic and scoping review recommendations that suggest internal and anticipated stigma may be addressed effectively with individual-level interventions like counseling or support groups, whereas enacted stigma may be better addressed with organizational or community-level information-based interventions [13, 63], all of which could be informed by survivor perspectives. Our IDIs further highlighted how stigma affects mental health, including experiences of depression and the long-term impact of TB on well-being. These findings align with others that underscore the importance of integrated mental health and TB services [64] and the need for implementation research on evidence-based interventions for co-morbid depression among PWTB.

Strengths of this study include the use of a validated stigma assessment tool [14] in two geographically distant communities with different cultural characteristics and a mixed methods approach that enabled triangulation of quantitative and qualitative data, enhancing the validity and credibility of our findings. The use of community-based participatory research that included TB survivors as PRAs and mutual ownership of this project by a TB advocacy organization fosters research equity, and maximizes the engagement of affected communities, and the relevance of the work for real-world implementation [65]. Study limitations include lower numbers of participants than planned due to the impact of the COVID-19 pandemic on study staffing and enrollment, limiting our ability to explore contextual differences in stigma between our study sites. Potential selection bias due to sampling facilitated by CHW referrals is possible but it is uncertain whether this could have led to people who experienced higher versus lower stigma being included. Given that the majority of our survey and IDI participants were women, we acknowledge selection bias with respect to gender, since men represent 62% of PWTB in South Africa [66]. This likely limits our ability to further delve into gender-based dimensions of stigma.

## Conclusions

Multi-component and multi-level approaches are needed to address the different experiences of stigma that additively and synergistically impede engagement in TB services. Interventions must incorporate contextual differences that may arise due to gender or setting, address HIV and other intersectional stigmas, and incorporate messaging from survivors to foster stigma resilience. TB stigma should be measured as an outcome, rather than merely considered as an intermediate step to improving programmatic outcomes. Community-engaged, evidence-based approaches to address stigma must be a priority for TB elimination efforts.

## Supplementary Information

Supplementary Material 1: Table S1: Stigma survey questions and responses, categorized according to stigma experiences for people who had TB and caregivers.

Supplementary Material 2. Use MY Voice to EndTB: empowering community health workers to destigmatize TB care in South Africa. Participant Interview Guide.

Supplementary Material 3. COREQ checklist.

Supplementary Material 4. Stigma survey results for caregivers.

Supplementary Material 5: Table S2. Study participant characteristics for caregivers of children with TB.

Supplementary Material 6: Table S3 Caregiver experiences of stigma.

Supplementary Material 7: Figure S1: Impact of anticipated, internal, and enacted stigma on care cascade engagement according to caregivers.

Supplementary Material 8: Table S4: Factors associated with stigma for caregivers of children who experienced TB.

## Data Availability

All data are freely available at Nathavitharana, Ruvandhi, 2024: “UseMyVoice to EndTB mixed methods stigma assessment data”, 10.7910/DVN/OEW8X9 Harvard Dataverse, V1.

## Abbreviations

CHW: Community Health Worker
CARD: Constraints, Actions, Risks or Desires
DR-TB: Drug-Resistant TB
HIV: Human Immunodeficiency Virus
IDI: In-depth interview
MDR-TB: Multidrug-resistant TB
PWTB: People with TB
PRA: Peer Research Associate
SASSA: South African Social Security Agency
TB: Tuberculosis
WHO: World Health Organization
XDR-TB: Extensively drug-resistant TB

## References

[1] World Health Organisation. Global Tuberculosis Report. Geneva, Switzerland; 2022 27 Oct 2022.

[2] Murray EJ, Bond VA, Marais BJ, Godfrey-Faussett P, Ayles HM, Beyers N. High levels of vulnerability and anticipated stigma reduce the impetus for tuberculosis diagnosis in Cape Town. South Africa Health policy and planning. 2013;28(4):410–8. 22945548 10.1093/heapol/czs072

[3] Mason PH, Roy A, Spillane J, Singh P. Social, historical and cultural dimensions of tuberculosis. J Biosoc Sci. 2016;48(2):206–32. 25997539 10.1017/S0021932015000115

[4] DeSanto D, Velen K, Lessells R, Makgopa S, Gumede D, Fielding K, et al. A qualitative exploration into the presence of TB stigmatization across three districts in South Africa. BMC Public Health. 2023;23(1):504. 36922792 10.1186/s12889-023-15407-2 PMC10017062

[5] Juniarti N, Evans D. A qualitative review: the stigma of tuberculosis. J Clin Nurs. 2011;20(13–14):1961–70. 21040040 10.1111/j.1365-2702.2010.03516.x

[6] Courtwright A, Turner AN. Tuberculosis and stigmatization: pathways and interventions. Public Health Rep. 2010;125(4_suppl):34–42. 10.1177/00333549101250S407 PMC2882973 20626191

[7] Stangl AL, Earnshaw VA, Logie CH, van Brakel W, C. Simbayi L, Barré I, et al. The Health Stigma and Discrimination Framework: a global, crosscutting framework to inform research, intervention development, and policy on health-related stigmas. BMC Medicine. 2019;17(1):31. 10.1186/s12916-019-1271-3 PMC6376797 30764826

[8] Broekmans J, Migliori G, Rieder H, Lees J, Ruutu P, Loddenkemper R, et al. European framework for tuberculosis control and elimination in countries with a low incidence: recommendations of the World Health Organization (WHO), International Union Against Tuberculosis and Lung Disease (IUATLD) and Royal Netherlands Tuberculosis Association (KNCV) Working Group. Eur Respir J. 2002;19(4):765–75. 11999007 10.1183/09031936.02.00261402

[9] Link BG, Phelan JC. Conceptualizing stigma. Ann Rev Sociol. 2001;27(1):363–85.

[10] United Nations. Political declaration of the UN general assembly high‐level meeting on the fight against tuberculosis. UNHQ New York; 2018.

[11] Uplekar M, Weil D, Lonnroth K, Jaramillo E, Lienhardt C, Dias HM, et al. WHO’s new end TB strategy. Lancet. 2015;385(9979):1799–801. 25814376 10.1016/S0140-6736(15)60570-0

[12] Nuttall C, Fuady A, Nuttall H, Dixit K, Mansyur M, Wingfield T. Interventions pathways to reduce tuberculosis-related stigma: a literature review and conceptual framework. Infect Dis Poverty. 2022;11(1):101. 36138434 10.1186/s40249-022-01021-8 PMC9502609

[13] Foster I, Galloway M, Human W, Anthony M, Myburgh H, Vanqa N, et al. Analysing interventions designed to reduce tuberculosis-related stigma: A scoping review. PLOS Global Public Health. 2022;2(10): e0000989. 36962638 10.1371/journal.pgph.0000989 PMC10022226

[14] Agnes Meershoek AZ, Amrita Daftary, Brian Citro, Caoimhe Smyth, Dean Lewis, Deirdre Ni Cheallaigh, Elaine Byrne, Ellen M. H Mitchell, Gill Craig, Ieva Leimane, James Malar, Jens Levy, Julia van der Land, Kate Macintyre, Lisa G. Johnston, Lisa Redwood, Nadine Ferris France, Nora Engel, Olive Mumba, Rajita Bhavaraju, Ronan R. Conroy, Sarah van de Berg, Stephen H-F Macdonald, Timur Abdullaev, and Tushar Nair. TB Stigma Measurement Guidance. The Hague, Netherlands; 2018 20 Aug 2018.

[15] Daniels J, Medina-Marino A, Glockner K, Grew E, Ngcelwane N, Kipp A. Masculinity, resources, and retention in care: South African men’s behaviors and experiences while engaged in TB care and treatment. Soc Sci Med. 2021;270: 113639. 33493956 10.1016/j.socscimed.2020.113639 PMC8445063

[16] Subbaraman R, Nathavitharana RR, Mayer KH, Satyanarayana S, Chadha VK, Arinaminpathy N, et al. Constructing care cascades for active tuberculosis: A strategy for program monitoring and identifying gaps in quality of care. PLoS Med. 2019;16(2): e1002754. 30811385 10.1371/journal.pmed.1002754 PMC6392267

[17] Naidoo P, Theron G, Rangaka MX, Chihota VN, Vaughan L, Brey ZO, et al. The South African tuberculosis care cascade: estimated losses and methodological challenges. J Infect Dis. 2017;216(suppl_7):S702-S13. 10.1093/infdis/jix335 PMC5853316 29117342

[18] Daftary A, Frick M, Venkatesan N, Pai M. Fighting TB stigma: we need to apply lessons learnt from HIV activism. BMJ Specialist Journals; 2017. p. e000515. 10.1136/bmjgh-2017-000515 PMC5717927 29225954

[19] Bresenham D, Kipp AM, Medina-Marino A. Quantification and correlates of tuberculosis stigma along the tuberculosis testing and treatment cascades in South Africa: a cross-sectional study. Infect Dis Poverty. 2020;9:1–13. 33092636 10.1186/s40249-020-00762-8 PMC7579945

[20] Creswell JW, Clark VP. Mixed methods research: Thousand Oaks: SAGE Publications; 2011.

[21] Ozawa S, Pongpirul K. 10 best resources on… mixed methods research in health systems. Health Policy Plan. 2014;29(3):323–7. 23564372 10.1093/heapol/czt019

[22] McDowell A, Engel N, Daftary A. In the eye of the multiple beholders: Qualitative research perspectives on studying and encouraging quality of TB care in India. J Clin Tuberc Other Mycobact Dis. 2019;16. 31497654 10.1016/j.jctube.2019.100111 PMC6716552

[23] Leung MW, Yen IH, Minkler M. Community based participatory research: a promising approach for increasing epidemiology’s relevance in the 21st century. Int J Epidemiol. 2004;33(3):499–506. 15155709 10.1093/ije/dyh010

[24] Carter A, Greene S, Nicholson V, O’Brien N, Sanchez M, de Pokomandy A, et al. Breaking the Glass Ceiling: Increasing the Meaningful Involvement of Women Living With HIV/AIDS (MIWA) in the Design and Delivery of HIV/AIDS Services. Health Care Women Int. 2015;36(8):936–64. 25256222 10.1080/07399332.2014.954703

[25] Ibáñez-Carrasco F, Watson JR, Tavares J. Supporting peer researchers: recommendations from our lived experience/expertise in community-based research in Canada. Harm Reduct J. 2019;16(1):1–5. 31481067 10.1186/s12954-019-0322-6 PMC6724244

[26] Greene S, Ahluwalia A, Watson J, Tucker R, Rourke SB, Koornstra J, et al. Between skepticism and empowerment: the experiences of peer research assistants in HIV/AIDS, housing and homelessness community-based research. Int J Soc Res Methodol. 2009;12(4):361–73.

[27] Kaida A, Carter A, Nicholson V, Lemay J, O’Brien N, Greene S, et al. Hiring, training, and supporting Peer Research Associates: Operationalizing community-based research principles within epidemiological studies by, with, and for women living with HIV. Harm Reduct J. 2019;16(1):47. 31319894 10.1186/s12954-019-0309-3 PMC6637632

[28] Fetters MD, Curry LA, Creswell JW. Achieving integration in mixed methods designs—principles and practices. Health Services Res. 2013;48(6pt2):2134–56. 10.1111/1475-6773.12117 PMC4097839 24279835

[29] Tuberculosis National Institute for Communicable Diseases, South Africa. National Health Laboratory Service M&E Online Dashboards. 2019 [cited 2023 October 5]. Available from: https://mstrweb.nicd.ac.za/Microstrategy/asp/Main.aspx.

[30] Partnership STB. TB Stigma Assessment Data Collection Instruments. Geneva: Switzerland; 2020.

[31] Harris PA, Taylor R, Minor BL, Elliott V, Fernandez M, O’Neal L, et al. The REDCap consortium: building an international community of software platform partners. J Biomed Inform. 2019;95: 103208. 31078660 10.1016/j.jbi.2019.103208 PMC7254481

[32] Hargreaves JR, Pliakas T, Hoddinott G, Mainga T, Mubekapi-Musadaidzwa C, Donnell D, et al. HIV Stigma and Viral Suppression Among People Living With HIV in the Context of Universal Test and Treat: Analysis of Data From the HPTN 071 (PopART) Trial in Zambia and South Africa. J Acquir Immune Defic Syndr. 2020;85(5):561–70. 32991336 10.1097/QAI.0000000000002504 PMC7654947

[33] Braun VCV. Using thematic analysis in psychology. Qual Res Psychol. 2006;3:77.

[34] Guest G, Namey E, Chen M. A simple method to assess and report thematic saturation in qualitative research. PLoS One. 2020;15(5). 32369511 10.1371/journal.pone.0232076 PMC7200005

[35] Nathavitharana RR, van der Westhuizen A, van der Westhuizen H-M, Mishra H, Sampson A, Meintjes J, et al. “If I’ve got latent TB, I would like to get rid of it”: Derivation of the CARD (Constraints, Actions, Risks, and Desires) Framework informed by South African healthcare worker perspectives on latent tuberculosis treatment. PLoS One. 2021;16(8). 34407070 10.1371/journal.pone.0254211 PMC8372902

[36] Nathavitharana, Ruvandhi, 2024, "UseMyVoice to EndTB mixed methods stigma assessment data", 10.7910/DVN/OEW8X9, Harvard Dataverse, V1

[37] South Africa Social Security Agency. Disability grant, Cape Town, South Africa: South African Government.,; 2023 [cited 2023 15 September 2023]. Available from: https://www.gov.za/services/social-benefits/disability-grant.

[38] Medina-Marino A, Bezuidenhout D, Ngcelwane N, Cornell M, Wainberg M, Beyrer C, et al. Qualitative Identification of Intervention Preferences to Support Men’s Engagement and Retention in TB Care in South Africa. Am J Mens Health. 2022;16(5):15579883221129348. 36218175 10.1177/15579883221129349 PMC9558889

[39] Medina-Marino A KA, Daftary A, Preacher K. Multi-level and Intersectional Stigma and other Social Determinant Effects on TB Case Detection, Care, and Treatment Outcomes: The MISSED TB Outcomes Study [Internet]. NIH RePORTER Database. [cited 2023 Oct 5]. Available from: https://reporter.nih.gov/project-details/10533331.

[40] Craig G, Daftary A, Engel N, O’driscoll S, Ioannaki A. Tuberculosis stigma as a social determinant of health: a systematic mapping review of research in low incidence countries. Int J Infect Dis. 2017;56:90–100. 10.1016/j.ijid.2016.10.011 27810521

[41] Timire C, Pedrazzoli D, Boccia D, Houben RM, Ferrand RA, Bond V, et al. Use of a sustainable livelihood framework-based measure to estimate socioeconomic impact of tuberculosis on house-holds. Clin Infect Dis. 2023;77(5):761–7. 10.1093/cid/ciad273 PMC10495125 37132328

[42] Furin J, Loveday M, Hlangu S, Dickson-Hall L, le Roux S, Nicol M, et al. “A very humiliating illness”: a qualitative study of patient-centered Care for Rifampicin-Resistant Tuberculosis in South Africa. BMC Public Health. 2020;20(1):1–11. 31952494 10.1186/s12889-019-8035-z PMC6969445

[43] Asabor EN, Vermund SH. Confronting structural racism in the prevention and control of tuberculosis in the United States. Clin Infect Dis. 2021;73(9):e3531–5. 33242078 10.1093/cid/ciaa1763 PMC8563219

[44] Teo AKJ, Tan RKJ, Smyth C, Soltan V, Eng S, Ork C, et al., editors. Characterizing and measuring tuberculosis stigma in the community: a mixed-methods study in Cambodia. Open forum infectious diseases; 2020: Oxford University Press US. 10.1093/ofid/ofaa422 PMC7585330 33134412

[45] Oladele DA, Balogun MR, Odeyemi K, Salako BL. A comparative study of knowledge, attitude, and determinants of tuberculosis-associated stigma in rural and urban communities of Lagos State, Nigeria. Tuberc Res Treat. 2020;2020:1–14. 10.1155/2020/1964759 PMC7728486 33343936

[46] Bergman A, Farley JE, Agarwalla V, Relf M. Reframing Intersectional Stigma for a South African Context Integrating Tuberculosis, HIV and Poverty Stigmas. J Assoc Nurses AIDS Care. 2022;33(1):22–32.34939985 10.1097/JNC.0000000000000296

[47] Van Schalkwyk C, Dorrington RE, Seatlhodi T, Velasquez C, Feizzadeh A, Johnson LF. Modelling of HIV prevention and treatment progress in five South African metropolitan districts. Sci Rep. 2021;11(1):5652. 33707578 10.1038/s41598-021-85154-0 PMC7952913

[48] Kerkhoff AD, Mwamba C, Pry JM, Kagujje M, Nyangu S, Mateyo K, et al. A mixed methods study on men’s and women’s tuberculosis care journeys in Lusaka, Zambia—Implications for gender-tailored tuberculosis health promotion and case finding strategies. PLOS Global Public Health. 2023;3(6): e0001372. 37327200 10.1371/journal.pgph.0001372 PMC10275452

[49] Department of Health, Republic of South Africa. National Guidelines on the Treatment of Tuberculosis Infection. Cape Town, South Africa; 2023.

[50] Stangl AL, Earnshaw VA, Logie CH, Van Brakel W, C. Simbayi L, Barré I, et al. The Health Stigma and Discrimination Framework: a global, crosscutting framework to inform research, intervention development, and policy on health-related stigmas. BMC Med. 2019;17:1–13. 10.1186/s12916-019-1271-3 PMC6376797 30764826

[51] Datiko DG, Jerene D, Suarez P. Stigma matters in ending tuberculosis: Nationwide survey of stigma in Ethiopia. BMC Public Health. 2020;20:1–10. 32028914 10.1186/s12889-019-7915-6 PMC7006204

[52] Ngamvithayapong-Yanai J, Luangjina S, Thawthong S, Bupachat S, Imsangaun W. Stigma against tuberculosis may hinder non-household contact investigation: a qualitative study in Thailand. Public Health Action. 2019;9(1):15–23. 30963038 10.5588/pha.18.0055 PMC6436487

[53] Nagarajan K, Muniyandi M, Sellappan S, Karunanidhi S, Senthilkumar K, Palani B, et al. A study on tuberculosis disease disclosure patterns and its associated factors: Findings from a prospective observational study in Chennai. PLoS One. 2023;18(1). 36701386 10.1371/journal.pone.0280812 PMC9879515

[54] Turan B, Hatcher AM, Weiser SD, Johnson MO, Rice WS, Turan JM. Framing mechanisms linking HIV-related stigma, adherence to treatment, and health outcomes. Am J Public Health. 2017;107(6):863–9. 28426316 10.2105/AJPH.2017.303744 PMC5425866

[55] Viljoen L, Wademan D, Hoddinott G, Bond V, Seeley J, Bock P, et al. The act of telling: South African women’s narratives of HIV status disclosure to intimate partners in the HPTN 071 (PopART) HIV prevention trial. Womens Health. 2021;17:1745506521998204. 10.1177/1745506521998204 PMC7924004 33645374

[56] Hlongwa M, Cornell M, Malone S, Pitsillides P, Little K, Hasen N. Uptake and short-term retention in HIV treatment among men in South Africa: The Coach Mpilo pilot project. Global Health: Science and Practice. 2022;10(1):e2100498. 10.9745/GHSP-D-21-00498. 10.9745/GHSP-D-21-00498 PMC8885359 35294387

[57] Hopkins KL, Hlongwane KE, Otwombe K, Dietrich J, Jaffer M, Cheyip M, et al. Does peer-navigated linkage to care work? A cross-sectional study of active linkage to care within an integrated non-communicable disease-HIV testing centre for adults in Soweto, South Africa. PLoS ONE. 2020;15(10): e0241014. 33091093 10.1371/journal.pone.0241014 PMC7580918

[58] Geng EH, Odeny TA, Montoya LM, Iguna S, Kulzer JL, Adhiambo HF, et al. Adaptive strategies for retention in care among persons living with HIV. NEJM Evidence. 2023;2(4):EVIDoa2200076. 10.1056/evidoa2200076 PMC10745095 38143482

[59] Daniels J, Medina-Marino, A. Assessing the Feasibility of Coach Mpilo for Men with TB and HIV in Eastern Cape, South Africa. NIH RePORTer. 2023.

[60] Ayakaka I, Armstrong-Hough M, Hannaford A, Ggita JM, Turimumahoro P, Katamba A, et al. Perceptions, preferences, and experiences of tuberculosis education and counselling among patients and providers in Kampala, Uganda: A qualitative study. Glob Public Health. 2022;17(11):2911–28. 35442147 10.1080/17441692.2021.2000629 PMC11005908

[61] Mwansa-Kambafwile JR, Jewett S, Chasela C, Ismail N, Menezes C. Initial loss to follow up of tuberculosis patients in South Africa: perspectives of program managers. BMC Public Health. 2020;20:1–8. 32375743 10.1186/s12889-020-08739-w PMC7201771

[62] Mntlangula MN, Khuzwayo N, Taylor M. Nurses perceptions about their behavioural counselling for HIV/AIDS, STIs and TB in eThekwini Municipality clinics KwAZulu-Natal. South Africa Health SA Gesondheid. 2017;22:52–60.

[63] Sommerland N, Wouters E, Mitchell EMH, Ngicho M, Redwood L, Masquillier C, et al. Evidence-based interventions to reduce tuberculosis stigma: a systematic review. Int J Tuberc Lung Dis. 2017;21(11):81–6. 29025489 10.5588/ijtld.16.0788

[64] Hayward SE, Deal A, Rustage K, Nellums LB, Sweetland AC, Boccia D, et al. The relationship between mental health and risk of active tuberculosis: a systematic review. BMJ Open. 2022;12(1): e048945. 34992103 10.1136/bmjopen-2021-048945 PMC8739435

[65] Key KD, Furr-Holden D, Lewis EY, Cunningham R, Zimmerman MA, Johnson-Lawrence V, et al. The continuum of community engagement in research: a roadmap for understanding and assessing progress. Prog Community Health Partnersh. 2019;13(4):427–34. 31866597 10.1353/cpr.2019.0064

[66] Cornell M, Horton K, Colvin C, Medina-Marino A, Dovel K. Perpetuating gender inequity through uneven reporting. Lancet. 2020;395(10232):1258. 32305091 10.1016/S0140-6736(20)30216-6 PMC8097709

