# Supplementary material for: “This is an illness. No one is supposed to be treated badly”: community-based stigma assessments in South Africa to inform tuberculosis stigma intervention design
Source: BMC Glob Public Health. 2024 Jun 24;2:41. doi: 10.1186/s44263-024-00070-5 (PMC11194205; doi:10.1186/s44263-024-00070-5)
Supplement: Supplementary file 1 — Supplementary Material 1: Table S1: Stigma survey questions and responses, categorized according to stigma experiences for people who had TB and caregivers. [file 44263_2024_70_MOESM1_ESM.docx]

**Additional file 1: Table S1: Stigma survey questions and responses, categorized according to stigma experiences for people who had TB and caregivers.**

|  | **Anticipated Stigma** | | | | | | | | | | |
| --- | --- | --- | --- | --- | --- | --- | --- | --- | --- | --- | --- |
|  | **People who had TB (adults) (n=93)** | | | | |  | **Caregivers of children (0-17 years) (n=24)** | | | | |
|  | Strongly Agree | Agree | Neutral | Disagree | Strongly Disagree |  | Strongly Agree | Agree | Neutral | Disagree | Strongly Disagree |
| I have worried that people might talk badly/gossiped about me/them because I/they had TB. * | 39% | 31% | 1% | 24% | 5% |  | 38% | 13% | 0% | 50% | 0% |
| I have worried that I/they would lose respect/standing in the community if people found out that I/they had TB. * | 27% | 38% | 0% | 29% | 6% |  | 29% | 13% | 0% | 58% | 0% |
| I have worried that I/they would be undermined by people if they found out I/they had TB. * | 20% | 45% | 1% | 29% | 4% |  | 25% | 13% | 0% | 54% | 4% |
| I have worried that people might hurt my/their feelings if they found out that I/they was living with TB. * | 23% | 44% | 1% | 28% | 4% |  | 25% | 21% | 0% | 54% | 0% |
| I have worried that I/they might lose friends if they found out I/they was living with TB. ^#^ | 16% | 41% | 2% | 33% | 6% |  | 29% | 13% | 4% | 54% | 0% |
| I/they have kept a distance from others to avoid spreading TB germs. * | 26% | 47% | 0% | 24% | 3% |  | 33% | 13% | 0% | 54% | 0% |
| I/they thought about avoiding going to TB clinics because other people might see me/them there. * | 18% | 34% | 0% | 34% | 12% |  | 17% | 4% | 0% | 67% | 13% |
| I worried about telling my family that I/they have TB because of what they might think about me/them. ^#^ | 13% | 32% | 1% | 43% | 11% |  | 21% | 8% | 0% | 63% | 8% |
| I worried about telling others that I/they have TB because they may think that I/they also have HIV/AIDS. * | 5% | 8% | 0% | 5% | 1% |  | 17% | 4% | 0% | 17% | 4% |
| I/they have felt guilty worrying about my family having the burden of caring for me/them. ^#^ | 22% | 42% | 0% | 30% | 5% |  | 21% | 4% | 0% | 75% | 0% |
| I have worried that if someone found out that I/they had TB they would think that it was because I/they smoke, drink, or do things that other people might think of as 'careless about my/their health'. * | 16% | 48% | 2% | 23% | 10% |  | 17% | 21% | 0% | 54% | 8% |
| My worries about TB stigma made me delay in recognizing [my child’s] TB symptoms. | 11% | 35% | 4% | 41% | 8% |  | 4% | 0% | 0% | 75% | 17% |
| My worries about TB stigma made me/them delay in going to the clinic. | 11% | 30% | 0% | 47% | 10% |  | 4% | 8% | 0% | 71% | 13% |
| My worries about TB stigma made me/them delay in starting treatment after being told that I/they have TB. | 3% | 22% | 0% | 57% | 17% |  | 4% | 4% | 0% | 79% | 13% |
| My worries about TB stigma made me/them delay in seeking out treatment adherence support. | 6% | 34% | 1% | 47% | 11% |  | 4% | 4% | 4% | 71% | 13% |
| My worries about TB stigma made me/them delay in completing treatment. | 2% | 18% | 2% | 61% | 16% |  | 4% | 4% | 4% | 67% | 17% |
| My worries about TB stigma made me/them delay in getting post-treatment follow-up services. | 5% | 17% | 2% | 60% | 15% |  | 4% | 13% | 0% | 71% | 13% |

*denotes experiences of stigma in the community

^#^ denotes experiences of stigma within the family

| **Internal Stigma** | | | | | | | | | | | |
| --- | --- | --- | --- | --- | --- | --- | --- | --- | --- | --- | --- |
|  | **People who had TB (adults) (n=93)** | | | | |  | **Caregivers of children (0-17 years) (n=24)** | | | | |
|  | Strongly Agree | Agree | Neutral | Disagree | Strongly Disagree |  | Strongly Agree | Agree | Neutral | Disagree | Strongly Disagree |
| I/my child have thought less of myself because I/they had TB. | 12% | 35% | 1% | 44% | 8% |  | 8% | 4% | 0% | 83% | 4% |
| I/my child have less respect for myself because I/they had TB. | 8% | 37% | 2% | 45% | 8% |  | 8% | 17% | 0% | 67% | 4% |
| I/my child have undermined myself by getting TB. | 6% | 35% | 2% | 46% | 10% |  | 13% | 25% | 0% | 58% | 4% |
| I/my child have felt that having TB means that I/they do not deserve to have friends. | 4% | 22% | 2% | 57% | 15% |  | 17% | 8% | 4% | 58% | 8% |
| I/my child have felt that having TB means I/they should be kept away from other people (alone). | 4% | 32% | 3% | 46% | 14% |  | 13% | 13% | 8% | 54% | 8% |
| I/my child have felt that people should keep distant from me/them to avoid TB germs. | 8% | 49% | 0% | 32% | 9% |  | 17% | 17% | 0% | 58% | 4% |
| I/my child have felt that I/they do not deserve care at the clinic. | 2% | 26% | 2% | 54% | 16% |  | 13% | 13% | 0% | 63% | 8% |
| I/my child have worried that I/they might have HIV/AIDS. | 4% | 8% | 0% | 6% | 1% |  | 4% | 4% | 0% | 21% | 8% |
| I/my child have become a burden for my/their family because of my/their TB. | 10% | 32% | 3% | 43% | 10% |  | 13% | 0% | 4% | 75% | 8% |
| I/my child probably got TB because I/they smoke, drink, or do other careless things. | 9% | 8% | 2% | 48% | 12% |  | 21% | 21% | 0% | 38% | 21% |
| My/my child’s negative thoughts about myself made me delay in recognizing TB symptoms. | 5% | 43% | 0% | 41% | 10% |  | 8% | 13% | 0% | 75% | 4% |
| My/my child’s negative thoughts about myself made me/them delay in going to the clinic. | 9% | 32% | 0% | 45% | 14% |  | 13% | 8% | 0% | 71% | 4% |
| My/my child’s negative thoughts about myself made me/them delay in starting treatment after being told that I/they have TB. | 1% | 19% | 1% | 54% | 22% |  | 4% | 13% | 0% | 79% | 4% |
| My/my child’s negative thoughts about myself made me/them delay in seeking out treatment adherence support. | 5% | 27% | 1% | 54% | 13% |  | 4% | 8% | 4% | 71% | 8% |
| My/my child’s negative thoughts about myself made me/them delay in completing treatment. | 1% | 19% | 0% | 61% | 17% |  | 4% | 13% | 0% | 79% | 4% |
| My/my child’s negative thoughts about myself made me/them delay in getting post-treatment follow-up services. | 1% | 19% | 0% | 59% | 19% |  | 4% | 4% | 4% | 83% | 4% |

| **Enacted Stigma** | | | | | | | | | | | |
| --- | --- | --- | --- | --- | --- | --- | --- | --- | --- | --- | --- |
|  | **People who had TB (adults) (n=93)** | | | | |  | **Caregivers of children (0-17 years) (n=24)** | | | | |
|  | Strongly Agree | Agree | Neutral | Disagree | Strongly Disagree |  | Strongly Agree | Agree | Neutral | Disagree | Strongly Disagree |
| People have talked badly/gossiped about me/my child because I/they had TB. * | 33% | 37% | 2% | 24% | 4% |  | 25% | 13% | 4% | 54% | 4% |
| I/my child have lost respect/standing in the community because I/they had TB. * | 20% | 31% | 1% | 39% | 8% |  | 17% | 0% | 8% | 71% | 4% |
| I/my child have been undermined by people because I/they had TB. * | 19% | 31% | 3% | 39% | 8% |  | 17% | 13% | 4% | 63% | 4% |
| People have made me/my child feel hurt by the way they reacted when they found out that I/they was living with TB. * | 16% | 35% | 4% | 35% | 8% |  | 13% | 17% | 8% | 50% | 8% |
| I/my child have lost friends when they found out I/they am living with TB. ^#^ | 10% | 19% | 2% | 58% | 9% |  | 17% | 8% | 0% | 63% | 13% |
| People have kept a distance from me/my child to avoid catching TB germs. * | 26% | 10% | 29% | 52% | 10% |  | 17% | 8% | 4% | 67% | 4% |
| People have seen me/my child at the TB clinic and therefore learned my/their status without me wanting to disclose to them. * | 29% | 34% | 2% | 28% | 6% |  | 17% | 4% | 8% | 63% | 8% |
| I/my child told someone that I/they had TB and they thought I/they also have HIV/AIDS. * | 2% | 6% | 0% | 8% | 3% |  | 13% | 8% | 4% | 13% | 4% |
| My family have told me or suggested that my [child’s] TB is a burden for them. ^#^ | 6% | 24% | 1% | 52% | 16% |  | 17% | 4% | 4% | 63% | 8% |
| Someone has told me that I/my child have TB because I/they smoke, drink, or do things that other people might think of as 'careless'. * | 4% | 24% | 1% | 56% | 13% |  | 13% | 8% | 0% | 71% | 8% |
| I have hidden my / my child’s TB treatment to avoid stigma. | 12% | 30% | 0% | 44% | 14% |  | 8% | 4% | 0% | 67% | 21% |
| Your child has hidden his/her TB treatment to avoid stigma | - | - | - | - | - |  | 8% | 4% | 0% | 71% | 17% |
| People stigmatizing me/my child made me delay in recognizing TB symptoms. | 5% | 33% | 2% | 48% | 11% |  | 4% | 4% | 4% | 83% | 4% |
| People stigmatizing me/my child made me delay in going to the clinic. | 6% | 34% | 0% | 47% | 11% |  | 4% | 4% | 4% | 79% | 4% |
| People stigmatizing me/my child made me delay in starting treatment after being told that I/they have TB. | 2% | 19% | 0% | 60% | 18% |  | 4% | 4% | 0% | 88% | 4% |
| People stigmatizing me/my child made me delay in seeking out treatment adherence support. | 2% | 27% | 1% | 56% | 14% |  | 4% | 4% | 0% | 88% | 4% |
| People stigmatizing me/my child made me delay in completing treatment. | 2% | 12% | 1% | 62% | 20% |  | 4% | 4% | 0% | 88% | 4% |
| People stigmatizing me/my child made me delay in getting post-treatment follow-up services. | 3% | 14% | 1% | 59% | 23% |  | 4% | 4% | 0% | 88% | 4% |

*denotes experiences of stigma in the community

^#^ denotes experiences of stigma within the family
