# Supplementary material for: “This is an illness. No one is supposed to be treated badly”: community-based stigma assessments in South Africa to inform tuberculosis stigma intervention design
Source: BMC Glob Public Health. 2024 Jun 24;2:41. doi: 10.1186/s44263-024-00070-5 (PMC11194205; doi:10.1186/s44263-024-00070-5)
Supplement: Supplementary file 2 — Supplementary Material 2. Use MY Voice to EndTB: empowering community health workers to destigmatize TB care in South Africa. Participant Interview Guide. [file 44263_2024_70_MOESM2_ESM.docx]

**Additional file 2: Use MY Voice to EndTB: empowering community health workers to destigmatize TB care in South Africa**

**Briefing for interview.**

[Introduction of the interviewer]

*Thank you for agreeing to take part. This interview is for a research study about understanding the experience of both those with TB and the carers or family members of those with TB. We greatly value your perspective. There are no right or wrong answers to the questions we will ask.*

*I would like to digitally record our conversation using this device. This will allow us to type our conversation, but any names or places that you mention will be taken out so that you will be anonymous. This way, if someone were to read this interview, they would not be able to know who you are because your name will be replaced with a code. If at any point during the interview you do not wish to answer a question that is ok.*

*Do you have any questions?*

**Topics**

Below is a list of topics to be discussed during the in-depth interviews. The questions will remain flexible to the participants’ agendas, and new topics may be added as the interviews progress. The key topic of stigma experienced by TB survivors and their carers will remain central throughout the interview.

1. Participant’s anticipations of stigma prior to, during or after their diagnosis and treatment of TB

2. Participant’s experiences of enacted stigma prior to, during or after their diagnosis and treatment of TB

3. Participant’s experiences of internalized of TB related stigma prior to, during or after their diagnosis and treatment of TB

Please note that this interview guide will be refined based on feedback from stakeholder discussions. The following are examples of questions that could be used. Given this study’s methodology (semi-structured interview), additional questions may be added during interviews following the topics listed above.

**Topic Area 1 - Personal introduction**

Can you tell me a bit about yourself:

How old are you? What is the gender you identify as? Who do you live with? Do you work- if so, what kind of work do you do?

**Topic Area 2 - Questions about general perceptions of TB stigma**

How are people with TB perceived in the community?

How are people with TB perceived within the healthcare system?

Why do you think people with TB may be treated differently from others?

Are people open to disclosing their TB status a) to their close contacts and b) more broadly to others in their community?

**Topic Area 3 - Illness narrative – to include questions re: experiences of stigma (enacted, internalized and anticipated)**

How did you find out that you had TB? Tell us more about your TB care journey?

- *Allow participant to share their TB story*

- *Tell me about times along this journey when you experienced stigma*

How did stigma affect your decision to seek care when you had first symptoms and then where you received your TB care?

Did your knowledge of other people’s experiences of TB affect your behaviour or decisions?

What major changes in your life took place because of TB?

How do you think these were affected by stigma?

- *Can prompt with example e.g. at the time of diagnosis versus during treatment versus post TB*

Can you tell us about your experience of TB care within the healthcare system and how this was affected by stigma?

Are people with TB treated differently if they are doing well on treatment versus if they are struggling with treatment?

**Topic area 4 - Experiences of stigma by domain**

Now please think about a time when you have experienced **enacted** stigma - this means when someone else did something to you or treated you badly or said something nasty because of your TB. What was the situation? Please describe what happened and how you experienced that.

- *Interviewer should have an example prepared in case participant needs prompting e.g. did anyone in the clinic or in the community behave differently towards you because you had TB?*

Now please think about a time when you have experienced **anticipated** stigma - this means when you thought someone **might** do something to you or treat you badly or said something nasty because of your TB. What was the situation? Please describe what happened and how you experienced that.

- *Interviewer should have an example prepared in case participant needs prompting e.g. did you worry that people in the clinic or in the community* ***might*** *behave differently towards you because you had TB or were you worried that people might think differently about you if they saw you at a TB clinic?*

Now please think about a time when you have experienced **internalized** stigma - this means when **you treated yourself badly or thought negative things about yourself** because of your TB. What was the situation? Please describe what happened and how you experienced that.

- *Interviewer should have an example prepared in case participant needs prompting e.g. did you feel badly about yourself in any way related to your TB diagnosis*

**Topic area 5 - recommendations for stigma reduction/mitigation/prevention**

Thank you very much for your time, we are doing this because we want to find ways to reduce TB stigma based on experiences like yours.

What I’ve heard from you is that ...

Putting that all together, how do you think we can reduce TB stigma in this community?

How would you change TB health services to reduce TB-related stigma?

**Tips:**

- Summarise what a person said and pause- then it creates space for them to add detail or correct you if you missed their message. If you want someone to elaborate you can take their sentence and say it as a question e.g. People called you names?

- Can you tell me about a time when you experienced xyz- ask for details about context “hang on, were you at home, was your brother with you?” then you get more for the story as it adds concrete information/ data.

- When people say something interesting, make a short note of it, so that you can perhaps add your questions in response to them e.g. ‘people see you going to the clinic and assume you have TB.’ Then you can ask, what about clinic visits make people assume you have TB, is it because of the frequent visits or because you are sent to a special area OR You said people don’t tell others that they have TB, why is that?

- We want it to feel like a conversation, do not have to follow it as a questionnaire. Build in pauses - add a prompt: reflect on what they said.

- Need to also remember our patients might have or have had intense experiences e.g. feeling suicidal that require a pause and check in to see if they are ok to continue or suggest mental health referral.

**Additional possible prompts to be used with topic areas above**

Questions re: Diagnosis

Can you tell me how you came to be diagnosed with TB?

Who did you talk to about your symptoms?

Who gave you your TB diagnosis?

Was there any counseling offered? What would you have liked to know at that time?

How did you feel when you were diagnosed with TB?

Who did you tell about your diagnosis?

Why did you choose these people to disclose to?

How did others make you feel when you were diagnosed with TB?

Questions re: Treatment

Did you tell people when you were receiving treatment for TB? If not, why?

How did people treat you upon learning that you were taking treatment for TB?

How did your own feelings about your TB diagnosis affect your experience of taking TB treatment?

How did other peoples’ perceptions about your TB diagnosis affect your experience of taking TB treatment?

What aspects of TB care would you change based on your experience?

Questions re: support

Who and what things helped you during your experience with TB?

What kind of support did you have from your family or community?

What kind of support did you have from the health system?

Post TB

How do you feel about yourself now that you have completed TB treatment?

Do you disclose to others that you have had TB? If not, why?

How has having TB impacted your life? What has changed/what do you do differently?

**Discussion guide for carers of people with TB**

**Topic Area 1 - Personal introduction**

Can you tell me a bit about yourself:

How old are you? What is the gender you identify as? Who do you live with? Do you work- if so, what kind of work do you do?

**Topic Area 2 - Questions about anticipation and perception of TB stigma**

How are people with TB perceived in the community?

How are people with TB perceived within the healthcare system?

Why do you think people with TB may be treated differently from others?

Are people open to disclosing their TB status a) to their close contacts and b) more broadly to others in their community?

**Topic Area 3 - Illness narrative – to include questions re: actual experiences of stigma and internalization of stigma**

Can you tell me how your [family member] found out they had TB?

- *Allow participant to share their TB story*

- *Tell me about times along this journey when you/they experienced stigma*

How did stigma affect their decision to seek care when they had first symptoms and then where they received your TB care?

Did your knowledge of other people’s experiences of TB affect your behaviour or decisions?

What major changes in their life took place because of TB? How do you think these were affected by stigma?

- *Can prompt with example e.g. at the time of your diagnosis versus during treatment versus post TB*

Can you tell us about their/your shared experience of TB care within the healthcare system and how this was affected by stigma?

Are people with TB treated differently if they are doing well on treatment versus if they are struggling with treatment?

**Topic area 4 - Experiences of stigma by domain**

Now please think about a time when your family member or you have experienced **enacted** stigma - this means when someone else did something to you or treated you badly or said something nasty because your family member had TB. What was the situation? Please describe what happened and how you experienced that.

- *Interviewer should have an example prepared in case participant needs prompting e.g. did anyone in the clinic or in the community behave differently towards you because your family member had TB?*

Now please think about a time when your family member or you have experienced **anticipated** stigma - this means when your family member or you thought someone **might** do something to you or treat you badly or said something nasty because your family member had TB. What was the situation? Please describe what happened and how you experienced that.

- *Interviewer should have an example prepared in case participant needs prompting e.g. did you worry that people in the clinic or in the community* ***might*** *behave differently towards you because you had TB or were you worried that people might think differently about you if they saw you at a TB clinic?*

Now please think about a time when you have experienced **internalized** stigma - this means when **your family member or you treated yourself badly or thought negative things about yourself** because your family member had TB. What was the situation? Please describe what happened and how you experienced that.

- *Interviewer should have an example prepared in case participant needs prompting e.g. did you feel badly about yourself in any way related to your TB diagnosis*

**Topic area 5 - recommendations for stigma reduction/mitigation/prevention**

Thank you very much for your time, we are doing this because we want to find ways to reduce TB stigma based on experiences like yours.

What I’ve heard from you is that ...

Putting that all together, how do you think we can reduce TB stigma in this community?

How would you change TB health services to reduce TB-related stigma?

**Prompt questions for carers of people with TB (targeted towards understanding stigma)**

**These can be used if narrative and questions above do not yield much discussion about stigma**

Questions re: diagnosis of family member with TB

How did they come to be diagnosed with TB?

Can you tell us about their experience of seeking care?

How did you feel when they started to have symptoms and when they were diagnosed with TB?

How did you feel about the implications of their TB diagnosis for your family?

Questions re: Treatment

Did you tell other people that your family member had TB? If not, why?

How did people treat you upon learning that your family member had TB?

How did you feel when you were caring for them during this time?

What aspects of TB care would you change based on your experience?

Questions re: support

Who and what things helped your family during your family member’s experience with TB?

What kind of support did you have from your family or community?

What kind of support did you have from the health system?

Post TB

How do you feel about people with TB now that your family member has completed TB treatment?

Do you disclose to others that your family member had TB? If not, why?

How has your family member having TB impacted your life?
