# Supplementary material for: “This is an illness. No one is supposed to be treated badly”: community-based stigma assessments in South Africa to inform tuberculosis stigma intervention design
Source: BMC Glob Public Health. 2024 Jun 24;2:41. doi: 10.1186/s44263-024-00070-5 (PMC11194205; doi:10.1186/s44263-024-00070-5)
Supplement: Supplementary file 4 — Supplementary Material 4. Stigma survey results for caregivers. [file 44263_2024_70_MOESM4_ESM.docx]

**Additional file 3: Stigma survey results for caregivers**

### Survey population characteristics.

Surveys were administered to 24 (20.5% of total population surveyed) caregivers. Most caregivers were women (58%). Most children had pulmonary TB (22/24) and three children had DR-TB; fewer were living with HIV (13%) (Table S2).

### Stigma experiences.

While caregivers overall reported stigma less commonly than PWTB, the majority of caregivers reported some form of anticipated (96%), internal (100%), and enacted (79%) stigma, based on their agreement or strong agreement with at least one item measuring each of these forms of stigma (Table S3).

### Anticipated, internal, and enacted stigma impacted engagement throughout the care cascade.

Compared to PWTB, caregivers more often disagreed that stigma impacted care cascade engagement (Figure S1). Univariate analyses did not demonstrate differences between stigma scores reported by caregivers (Table S4).
