# Supplementary material for: “This is an illness. No one is supposed to be treated badly”: community-based stigma assessments in South Africa to inform tuberculosis stigma intervention design
Source: BMC Glob Public Health. 2024 Jun 24;2:41. doi: 10.1186/s44263-024-00070-5 (PMC11194205; doi:10.1186/s44263-024-00070-5)
Supplement: Supplementary file 5 — Supplementary Material 5: Table S2. Study participant characteristics for caregivers of children with TB. [file 44263_2024_70_MOESM5_ESM.docx]

**Additional file 4: Table S2.** **Study participant characteristics for caregivers of children with TB.**

|  | **Caregivers (n=24)** |
| --- | --- |
| ***Demographic Variables*** | |
| **Median Age (years, IQR)** | * |
| **Gender, n (%)**  Woman  Man | 14 (58)  10 (42) |
| **Language, n (%)**  English  Xhosa  Sotho  Missing | 4 (17)  20 (83)  0 (0)  0 (0) |
| **Location, n (%)**  Khayelitsha  Hammanskraal  Missing | 23 (96)  0 (0)  1 (4) |
| ***Clinical Variables*** | |
| **HIV, n (%)**  Yes  No  Refused  Missing | 3 (13)  8 (33)  2 (8)  11 (46) |
| **TB Type, n (%)**  Pulmonary  Extra-pulmonary | 22 (92)  2 (8) |
| **Drug Resistance Type, n (%)**  Drug Sensitive  MDR or XDR | 21 (87)  3 (13) |

Abbreviations: IQR= Interquartile Range, SD= Standard Deviation, MDR=Multidrug Resistant, XDR= Extensively Drug Resistant

*Age was often inconsistently recorded as the age of the caregiver instead of the child thus not reported here.
