# Supplementary material for: “This is an illness. No one is supposed to be treated badly”: community-based stigma assessments in South Africa to inform tuberculosis stigma intervention design
Source: BMC Glob Public Health. 2024 Jun 24;2:41. doi: 10.1186/s44263-024-00070-5 (PMC11194205; doi:10.1186/s44263-024-00070-5)
Supplement: Supplementary file 6 — Supplementary Material 6: Table S3 Caregiver experiences of stigma. [file 44263_2024_70_MOESM6_ESM.docx]

**Additional file 5: Table S3** **Caregiver experiences of stigma.**

|  | **Caregivers (n=24)** |
| --- | --- |
| ***At least 1 survey item with an agree/strongly agree response*** |  |
| **Anticipated stigma (11 items), n (%)** | 23 (96) |
| **Internal stigma (10 items), n (%)** | 24 (100) |
| **Enacted stigma (11 items), n (%)** | 19 (79) |
| ***At least 5 items with an agree/strongly agree response*** |  |
| **Anticipated stigma (11 items), n (%)** | 12 (50) |
| **Internal stigma (10 items), n (%)** | 11 (46) |
| **Enacted stigma (11 items), n (%)** | 9 (38) |
