# Supplementary material for: “This is an illness. No one is supposed to be treated badly”: community-based stigma assessments in South Africa to inform tuberculosis stigma intervention design
Source: BMC Glob Public Health. 2024 Jun 24;2:41. doi: 10.1186/s44263-024-00070-5 (PMC11194205; doi:10.1186/s44263-024-00070-5)
Supplement: Supplementary file 7 — Supplementary Material 7: Figure S1: Impact of anticipated, internal, and enacted stigma on care cascade engagement according to caregivers. [file 44263_2024_70_MOESM7_ESM.docx]

Anticipated Internal Enacted


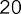

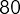

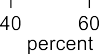

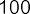

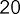

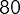

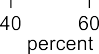

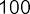

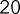

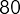

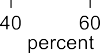

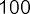


|  | | | | | | |
| --- | --- | --- | --- | --- | --- | --- |
|  |  | | | | |  |
|  | |  |  |  |  | |
|  |  | | | | |  |
|  | |  |  |  |  | |
|  | | | | | |  |
|  | |  |  |  |  | |
|  | | | | | |  |
|  | |  |  |  |  | |
|  | | | | | |  |
|  | |  |  |  |  | |
|  | | | | | |  |
|  | |  |  |  |  | |

Stigma delayed symptom recognition

Stigma delayed initial clinic visit Stigma delayed starting treatment

Stigma delayed seeking adherence support Stigma delayed completing treatment Stigma delayed seeking post-treatment care


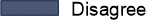

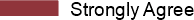

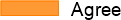

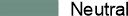


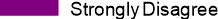
Additional file 6: Figure S1: Impact of anticipated, internal and enacted stigma on care cascade engagement according to caregivers.
