# Supplementary material for: “This is an illness. No one is supposed to be treated badly”: community-based stigma assessments in South Africa to inform tuberculosis stigma intervention design
Source: BMC Glob Public Health. 2024 Jun 24;2:41. doi: 10.1186/s44263-024-00070-5 (PMC11194205; doi:10.1186/s44263-024-00070-5)
Supplement: Supplementary file 8 — Supplementary Material 8: Table S4: Factors associated with stigma for caregivers of children who experienced TB. [file 44263_2024_70_MOESM8_ESM.docx]

**Additional file 7: Table S4: Factors associated with stigma for caregivers of children who experienced TB.**

|  | **Anticipated Stigma** | | **Internal Stigma** | | **Enacted Stigma** | |
| --- | --- | --- | --- | --- | --- | --- |
| **Variable** | Crude  β-coef (95% CI) | p-value | Crude  β-coef (95% CI) | p-value | Crude  β-coef (95% CI) | p-value |
| **Gender, n (%)**  Woman  Man | *Reference*  0.08 (-0.46, 0.62) | -  0.752 | *Reference*  -0.08 (-0.60, 0.44) | -  0.748 | *Reference*  0.31 (-0.40, 1.03) | -  0.375 |
| **HIV, n (%)***  Yes  No | *Reference*  -0.32 (-1.02, 0.38) | -  0.336 | *Reference*  -0.30 (-1.01, 0.42) | -  0.380 | *Reference*  -0.52 (-1.53, 0.50) | -  0.284 |
| **TB Type, n (%)**  Pulmonary  Extrapulmonary | *Reference*  0.10 (-0.87, 1.06) | -  0.838 | *Reference*  0.17 (-0.76, 1.10) | -  0.715 | *Reference*  0.42 (-0.86, 1.71) | -  0.501 |
| **Drug Resistance, n (%)**  Drug Sensitive  DR-TB | *Reference*  -0.45 (-1.23, 0.34) | -  0.249 | *Reference*  -0.31 (-1.08, 0.46) | -  0.412 | *Reference*  -0.08 (-1.16, 1.00) | -  0.879 |
